# Supplementary material for: Caffeic Acid Modulates Processes Associated with Intestinal Inflammation
Source: Nutrients. 2021 Feb 8;13(2):554. doi: 10.3390/nu13020554 (PMC7914463; doi:10.3390/nu13020554)
Supplement: Supplementary file 1 [file nutrients-13-00554-s001.pdf]

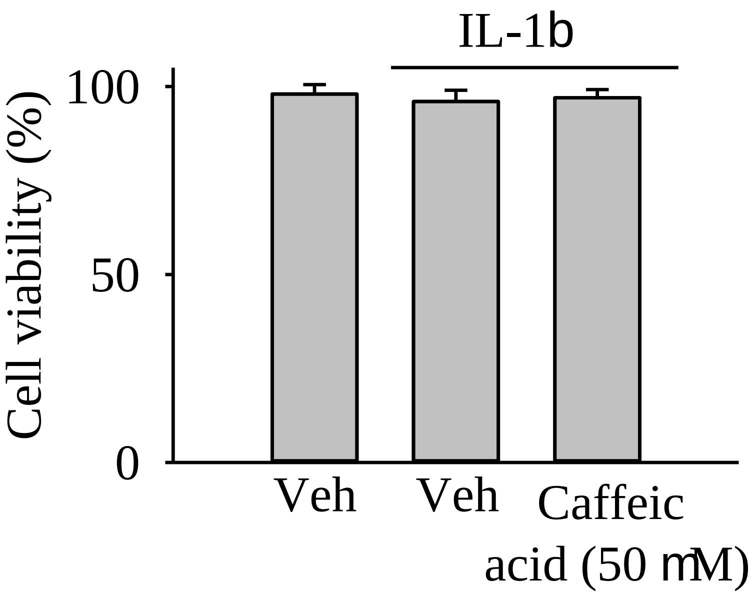

Supplementary Figure 1. Effect of caffeic acid and(or) 1ng/mL IL-1β on myofibroblasts of colon cell viability after 24 h of treatment. The results show the average ± S.D. of viable cells (%) obtained from three independent experiments (n=3).
